# Antiphospholipid syndrome; its implication in cardiovascular diseases: a review

**DOI:** 10.1186/1749-8090-5-101

**Published:** 2010-11-03

**Authors:** Ioanna Koniari, Stavros N Siminelakis, Nikolaos G Baikoussis, Georgios Papadopoulos, John Goudevenos, Efstratios Apostolakis

**Affiliations:** 1 Cardiothoracic Surgery Department. University of Patras, School of Medicine. Patras Greece; 2 Cardiac Surgery Department. University of Ioannina, School of Medicine. Ioannina Greece; 3 Department of Clinical Anaesthesiology and Intensive Postoperative Care Unit. University of Ioannina, School of Medicine. Ioannina Greece; 4 Cardiology Department. University of Ioannina, School of Medicine. Ioannina Greece

## Abstract

Antiphospholipid syndrome (APLS) is a rare syndrome mainly characterized by several hyper-coagulable complications and therefore, implicated in the operated cardiac surgery patient. APLS comprises clinical features such as arterial or venous thromboses, valve disease, coronary artery disease, intracardiac thrombus formation, pulmonary hypertension and dilated cardiomyopathy. The most commonly affected valve is the mitral, followed by the aortic and tricuspid valve. For APLS diagnosis essential is the detection of so-called antiphospholipid antibodies (aPL) as anticardiolipin antibodies (aCL) or lupus anticoagulant (LA). Minor alterations in the anticoagulation, infection, and surgical stress may trigger widespread thrombosis. The incidence of thrombosis is highest during the following perioperative periods: preoperatively during the withdrawal of warfarin, postoperatively during the period of hypercoagulability despite warfarin or heparin therapy, or postoperatively before adequate anticoagulation achievement. Cardiac valvular pathology includes irregular thickening of the valve leaflets due to deposition of immune complexes that may lead to vegetations and valve dysfunction; a significant risk factor for stroke. Patients with APLS are at increased risk for thrombosis and adequate anticoagulation is of vital importance during cardiopulmonary bypass (CPB). A successful outcome requires multidisciplinary management in order to prevent thrombotic or bleeding complications and to manage perioperative anticoagulation. More work and reporting on anticoagulation management and adjuvant therapy in patients with APLS during extracorporeal circulation are necessary.

## Introduction

Antiphospholipid syndrome (APLS) [1,2] comprises clinical features such as arterial or venous thromboses and the detection of so-called antiphospholipid antibodies (aPL) as anticardiolipin antibodies (aCL) or lupus anticoagulant (LA). APLS may be the most common acquired hypercoagulable state, occurring in up to 2% of the general population [3,4]. However, not all patients with these antibodies will develop the antiphospholipid syndrome, as antiphospholpid antibodies have been found in about 5% of the healthy population [5]. Patients with APLS have a significant involvement of the cardiovascular system. Coronary artery disease and valvular abnormalities constitute the most frequent manifestations representing more than two-thirds of cases [5]. Several studies have demonstrated that hypercoagulability of APLS patients predisposes to high rates of thromboembolic events as well as high rate of restenosis of the coronaries and the grafts after percutaneous interventions or CABG respectively, causing significant morbidity and mortality [6,7]. Especially, APLS patients can develop vasculo-occlusive complications before operation with the reversal of preoperative anticoagulation, intraoperatively due to inadequate anticoagulation during bypass and postoperatively before the achievement of adequate anticoagulation [8]. Therefore, the management of APLS patient can be quite challenging both for cardiologist and cardiac surgeon.

## Etiology-Pathophysiology

Anticardiolipin (aCL) antibodies are a heterogeneous family of auto-antibodies directed against protein-phospholipid complexes [6]. It is now generally accepted that there is a group of patients in whom high titers of aCL antibodies, usually the IgG class, and thrombotic features occur without clinical manifestations of systemic lupus erythematosus (SLE): primary APLS [2,6]. Anticardiolipin antibodies can be also observed in patients with SLE, or other autoimmune diseases (e.g. rheumatoid arthritis): secondary APLS. Moreover, it has been proved that the pathogenic antibodies accountable for the APLS main symptoms are not direct aPL against phospholipids itself; as produced in infections (e.g. syphilis), neoplastic disorders or induced by certain drugs (e.g. phenothiazines, quinidine) but rather indirect"aPL" directed against certain phospholipid depending proteins [2,9]. The targets of pathogenic antibodies in APLS are plasma or vascular cell proteins. Specifically, the main target antigens reported in patients with APLS include beta-2-glycoprotein-1 (b2GPI), prothrombin and annexin V [2,10]. Other putative antigens are thrombin, protein C, protein S, thrombomodulin, tissue plasminogen activator, kininogens (high or low molecular), prekallikrein, factor VII/VIIa, factor XI, factor XII, complement component C4, heparan sulfate proteoglycan, heparin, oxidised low-density lipoproteins [10,11]. The main autoantigens are attracted to negatively charged phospholipids (PL^(-)^) exposed on the outer side of cell membranes in great amounts only under special circumstances such as damage or apoptosis (e.g. endothelial cell) or after activation (e.g. platelets) [2,12]. Several membrane receptors have been recognized as signal transducers and after intracellular processing of the signal, the expression of adhesion molecules as E-selectin, vascular-cell-adhesion-molecule-1 (VCAM-1) or intracellular adhesion-molecule-1 (ICAM-1) increase the adhesion of immunocompetent cells further activating endothelial cells [2,13]. Furthermore, the production of tissue factor or inhibition of tissue-factor-pathway-inhibitor (TFPI) activates the extrinsic coagulation pathway [2,14], while the simultaneous decreased production of prostacyclin induces vasoconstriction and platelet aggregation. The activation of platelets results in the production of thromboxane A2 with further platelet activation and increased adhesion to collagen [15]. On the other hand, the displacement of tissue type plasminogen activator (t-PA) from annexin II, an endothelial cell membrane receptor and simultaneously enhancer to t-PA [16] could reduce the plasmin activation leading in deceleration of fibrinolysis [2]. The above potential activated pathways cause a prothrombotic state in APLS (table 1).

**Table 1:** Pathways and mechanisms resulting in a prothrombotic state in APLS

Pathway	Mechanism
Activation of endothelial cells:	expression of adhesion molecules or tissue factor (2,13,14)
Activation of thrombocytes:	induction of thromboxane A2, increased adhension (15)
Activation of coagulation cascade:	A. tissue factor production (activation of extrinsic pathway: monocytes (14)
	B. via thrombin activation (direct mechanism) (2,10)
	C. via cell activation (indirect mechanism) (2)
Inhibition of anticoagulation:	A. inhibition of plasminogen/plasmin (2,16)
	B. inhibition of t-PA by displacement from annexin II (16)
	C. inhibition of protein C by thrombomodulin (2,11)
	D. inhibition of protein S (11)

Generally, the binding of aPL to platelet membrane phospholipid-bound proteins may initiate platelet aggregation and thrombosis. Thrombosis may comprise the final common pathway of many processes, each based on its own particular autoantibody profile [8,17]. Indeed, in nearly 30% of patients with APLS, aPL antibodies react with phospholipids on the surface of activated platelets causing platelet adhension and thrombocytopenia. As only activated platelets expose phospholipid, it is usually thrombotic APLS patients who develop thrombocytopenia [18]. However, thrombocytopenia is not protective against thrombosis. Several explanations exist as to why increased aCL may contribute to increased thrombotic risk, including platelet damage, interference with antithrombin III activity, and inhibition of prekallikrein or protein C activation by thrombomodulin [19,20]. In addition, thrombotic complications appear to result from aPL-mediated displacement of annexin-V from phospholipid surfaces [21]. This displacement of annexin-V increases the quantity of coagulation factor binding sites potentially leading to a procoagulant state [22]. Because many individuals with high aPL antibody titers remain asymptomatic, several studies have proposed a 2-hit hypothesis. The presence of aPL antibodies induces endothelial dysfunction (first hit) and another condition (second hit) such as pregnancy infection, or vascular injury trigger thrombosis [8,23].

## Clinical manifestations of APLS

Cardiac manifestations in APLS include valvular disease, coronary artery disease, intracardiac thrombus formation, pulmonary hypertension and dilated cardiomyopathy [5,8]. Cardiac valvular pathology includes irregular thickening of the valve leaflets due to deposition of immune complexes that may lead to vegetations and valve dysfunction. These lesions are frequent and may be a significant risk factor for stroke [8]. Noninfectious and noninflammatory but rather thrombotic or fibrotic/calcific lesions are found in patients with primary APLS [2,24]. APLS is combined with SLE in about 40% of all APLS patients and consequently heart valvular lesions can also be caused by a SLE specific mechanism [24]. Non-autoimmunogenic reasons for heart valve failure in APLS-patients are possible as well [2]. The most commonly affected valve is the mitral, followed by the aortic and tricuspid valves; because the surface of the left-sided valves is more vulnerable to micro injuries due to stress, jet effect and turbulence [2,8]. Notably, the incidence of arterial embolization is estimated to be 77% in patients with APLS and simultaneous mitral valve disease [8,25]. Several studies have demonstrated a positive correlation between the aCL titers and valvular heart disease severity [5,7]. Most patients develop a mild form of valvular regurgitation while 4-6% of patients progress to severe valvular regurgitation necessitating replacement surgeries [5,26]. The rather young age of the patients and the most often necessary long-term anticoagulation for APLS seem to make a mechanical valve the first choice if a replacement is needed but thromboembolic complications render a mechanical valve in danger of dysfunction [2,27]. The advantage of a bioprosthesis is the independence of oral anticoagulation, however valve failure due to excessive pannus and consecutive stenosis renders replacement inevitable after some years [2,28]. Additionally, accelerated atherosclerosis increases the risk of coronary artery disease; the etiology seems to be more related with inflammatory and immunopathologic factors as compared with traditional Framingham cardiovascular risk factors [8,29]. The presence of intracardiac thrombus is a rare but potentially a life-threatening manifestation of APLS. Thrombus formation, a potential cause of pulmonary and systemic emboli, may occur in any cardiac chamber but most frequently on the right side [8]. Antiphospholipid antibodies have been associated with various other thrombogenic complications such as recurrent thrombosis, thrombocytopenia and serious bleeding abnormalities [30-32]. Of those patients with APLS who present with thrombosis, 30 ± 55% will present with venous thrombosis, especially of the lower limbs [18]. Repeat episodes of thrombosis are often of the same type [33]. However, it is paradox that despite the well-documented coexistence of autoantigens and its antibodies in blood of APLS patients for long periods, thrombotic events occur only sporadically. Eventually, it can be assumed that thrombotic events occur much more often but only in microvasculature or smaller vessels, resulting in deterioration of organ function such as renal failure, cerebral damages or impairment of the myocardial function by multiple recurrent microthromboses or microemboli [26]. Neurologic manifestations of APLS have been reported to include recurrent cerebral infarcts, headaches, migraines and visual disturbances [34,35]. Other manifestations of APLS include skin ulcers (pyoderma gangrenosum- like or livedoid vasculitis) due to fibrin deposition within the lumens of superficial dermal vessels as well as diffuse alveolar haemorrhage [36]. Of note, acute diffuse alveolar haemorrhage may be present in fully anticoagulated patients that could be attributed to a non-thrombotic pathogenesis in contrast to typical alveolar haemorrhage. Catastrophic APLS (cAPLS) is an acute condition with multiple vascular occlusions resulting in failure of several organs simultaneously or over a short period of time (days to weeks) [2,37]. It can be triggered by surgery, infection or changes in anticoagulation therapy [3,4]. cAPLS presents a mortality of 50% and can resemble syndromes such as heparin-induced thrombocytopenia (HIT), disseminated intravascular coagulation (DIC), systemic inflammatory response syndrome (SIRS), SLE vasculitis, thrombotic thrombocytopenic purpura (TTP) or sepsis [2].

## Diagnosis

Diagnosis of APLS includes clinical criteria of thrombosis and/or pregnancy morbidity and laboratory proof of lupus anticoagulants and/or anticardiolipin antibodies in medium or high titers on two or more occasions at least twelve weeks apart [2,25]. aPL antibodies are a heterogeneous group and thus diagnosis requires more than one test. The two main antibody groups are aCL antibodies and LA; however patients with aCL antibodies are five times more common than those with LA [18]. Results of aCL assays are expressed as IgG and IgM phospholipids units (GPL or MPL units) based on standard curves. According to the updated classification criteria for APLS [1] the diagnosis of APLS requires at least one of the following clinical (vascular thrombosis or complications of pregnancy) and one of the laboratory criteria.

### Diagnostic (classification) criteria of APLS (1)

#### I. Clinical criteria

##### 1. Vascular thrombosis

Arterial, venous or small vessel thrombosis in any tissue or organ, to be confirmed by objective validated criteria (imaging studies or histopathology). For histopathologic confirmation, thrombosis should be present without significant evidence of inflammation in the vessel wall.

##### 2. Pregnancy morbidity

-One or more unexplained deaths of a morphologically normal fetus beyond the 10th week of gestation or

-One or more premature births of a morphologically normal neonate before the 34th week of gestation because of eclampsia or preeclampsia or placental insufficiency.

-Three or more unexplained consecutive spontaneous abortions before the 10th week of gestation.

#### II. Laboratory criteria

1. Lupus anticoagulants in plasma.

2. Anticardiolipin antibody of IgG and/or IgM isotype in serum or plasma, present in medium or high titer (i.e. >40 GPL or MPL).

3. Anti-beta2Glycoprotein1-antibodies of IgG and/or IgM isotype in serum or plasma.

## Laboratory tests: approaching potential limitations

The definition of "APLS" requires the positive testing for lupus anticoagulant, anti-cardiolipin or anti-b2GPI twice at least 12 weeks apart [1]. The double testing contributes to the exclusion of patients with a transient reactivity by direct antiphospholipid-antibodies due to infections or other contributory factors [2,38]. "Lupus anticoagulant" is a misnomer for a group of various phospholipid inhibitors, observed usually without underlying SLE and in vivo related not to bleeding but to thrombotic complications. Coagulation tests used to reveal lupus anticoagulant include: activated partial thromboplastin time (aPTT), diluted Russell's viper venom test (dRVVT), taipan venom test, textarin venom/ecarin venom clotting time ratio, kaolin clotting time and tissue thromboplastin inhibition test [38]. Generally, tests are sensitive but usually need a meticulous interpretation. For example, a prolongation of clotting tests such as aPTT, usually is an indication of a bleeding tendency, but in APLS patients is correlated with a high risk for thrombotic/thromboembolic events [2,39]. Especially, some tests such as prothrombin time (PT), aPTT, or dRVVT based on both calcium and phospholipids adequacy for further activation of several clotting factors (Factor II, VII, IX, X) [40]. If the measured time of clot forming after addition of Ca2+ and PL^(-) ^is prolonged, a possible explanation is the existence of antibodies interacting with phospholipids. If the clotting time is not corrected by addition of normal plasma (mixing step - corrects missing coagulation factors) but tends to be normal by adding an excessive amount of phospholipids (confirmation step by adding activated thrombocytes) the LA effect is considered "positive" [2,29]. However, LA testing presents several drawbacks:

**a**. LA test, considered highly sensitive but not very specific concerning thromboembolic risk in APLS patients, can only be reliable if potential sources for phospholipids are removed before testing. In daily routine plasma for clotting tests is prepared by centrifugation of a blood sample, removing red and white blood cells. However, the smaller and lighter thrombocytes stay mainly in the supernatant providing a rich source of PL^(-) ^[2,38].

**b**. The prothrombin time is a routine test depending on Ca2+ and PL^(-) ^in factor VII, X and II [29]. The activator, tissue thromboplastin, is extracted from animal tissues bounding in PL^(-) ^, rendering this test useless for APLS diagnosis. Recently a modified test with an exact amount of recombinant TF and synthetic phospholipids [2,38] is available allowing the detection of LA.

**c**. aPTT test may differ because of different ingredients concerning its sensitivity towards the LA effect. Especially, the activator (e.g. kaolin, silica, ellagic acid or celite), the phospholipid source (several animal or plant sources) and finally the clot-detection method/instruments (photo-optical, mechanical, manual) can vary widely, influencing the test result [2,40]. Similar to aPTT is the principle of the dRVVT. At least, both these tests should always be performed when plasma is tested for an LA-effect [1]. Anti-cardiolipin test, the classic solid phase assay test, should be considered only as a differential diagnosis in a positive test result, because direct antibodies to CL are not pathogenic for APLS. One of test limitations, is that both patient's serum and buffer solution (usually bovine serum) provide homologous β2GPI, introducing an extra amount of target antigens [41]. Therefore, the test result can be influenced in case of a present human autoantibody (especially isotype IgM) that reacts exclusively with human b2GPI [2,41]. Fact that makes the colour reaction less powerful and false-negative as the strength of reaction is significant for APLS diagnosis; consequently only moderate to high titer antibodies are considered as "positive aCL" for APLS [1,2]. Moreover, as mentioned already for the LA-tests the plasma should be platelet depleted; otherwise exposed PL^(-) ^on the surface of activated platelets attract b2GP. A final pitfall of anti-cardiolipin test is that CL represents one of several negatively charged phospholipids rendering possible to miss a few cases in which specific autoantibodies react with b2GPI only when bound to another PL^(-) ^, e.g. phosphatidylserine [2]. Finally, anti-b2GPI and anti-prothrombin ELISA tests promise a more specific diagnosis of APLS. Beyond anti-b2GPI, ELISA tests for antiPT, with prothrombin directly fixed on a plate or via phosphatidylserine (antiPS-PT) add further information especially if tests for LA, aCL or anti-b2GPI are persistently negative [2,42]. It is debating if these tests have to be repeated for confirmation of the diagnosis APLS because they are theoretically not influenced by non-pathogenic antibodies, cancer or drugs [2,42].

## Relation of aCL with restenosis following percutaneous coronary interventions (PCI) in CAD patients

The role of serum aCL levels in natural history and prognosis of acute coronary syndromes (ACS) is still undetermined. However, anticardiolipin antibodies have been found to be associated with arterial and venous thrombosis [43]. Angioplasty-induced arterial injury leads to platelet aggregation, adhesion, and thrombosis. Several factors that are involved in the thrombogenesis may influence the restenosis rate after PTCA as thrombosis is one of the possible mechanisms of restenosis. Different mechanisms are associated with high aCL-IgG levels and restenosis after PCI [6]. Contradictory results have been demonstrated concerning the effect of aCL antibodies on restenosis. Eber, *et al *[44] showed that aCL-IgM was an independent risk factor for restenosis after PTCA in 65 men with coronary artery disease, however, no correlation was found between aCL-IgG and restenosis. Ludia, *et al *[45] reported that restenosis was more frequent in aCL positive patients with ischemic heart disease. Gurlek et al [6] studied the follow-up coronary angiography in two groups of 80 patients with acute coronary syndrome, in comparison to IgM and IgG aCL levels measured before hospital discharge. The results suggested that restenosis occurs more frequently in anticardiolipin positive patients. In contrast, Chiarugi, *et al *[46] observed no association between the presence of aCL and clinical restenosis, however, the presence of aCL with elevated lipoprotein a [Lp(a)] levels, acting synergistically, increased the risk of restenosis. Finally, in a recent study, Sharma S et al [47], failed to demonstrate any significant correlation between the level of IgG anticardiolipin antibodies and in-stent restenosis in patients having undergone PCI with bare metal or drug eluting stents. The corellation of elevated aCL levels and post-ACS cardiovascular events is still controversial. The largest study on this issue was recently reported by Bili et al, [48] who studied 1150 AMI patients, demonstrating that elevated aCL-IgG and low aCL-IgM antibodies were independent risk factors for recurrent cardiovascular events. Zuckerman et al, [49] suggested that the presence of aCL is a marker for increased risk for myocardial reinfarction and thromboembolic events after acute myocardial infarction (MI). However, Hamsten and colleagues [50] demonstrated that antibodies to cardiolipin are markers for a high risk of recurrent cardiovascular events in young survivors of MI; but their study was small in scope. On the contrary, Sletnes, et al [51] in 597 acute MI survivors, using multivariate analysis, failed to prove that aCL is an independent risk for mortality, cerebral thromboembolism, or recurrent MI. Analogous results were obtained by Cortellaro et al, [52] in their study of 74 young MI patients and Phadke et al, [53] who measured aCL in 299 survivors of acute MI. Eventually, Gurlek et al, [6] found no association between aCL antibodies with recurrent cardiovascular events (reinfarction and intracardiac thrombus formation) in ACS patients.

## Intraoperative management of coagulation: a crucial problem

Patients with APLS are at increased risk for thrombosis and adequate anticoagulation is of vital importance during cardiopulmonary bypass (CPB) [18,30,31,54,55]. Perioperative risks include thrombosis and/or bleeding secondary to excessive anticoagulation or APLS associated clotting factor deficiencies (especially factor II) [18]. In the meantime, minor alterations in anticoagulant therapy, infection, or a surgical insult may trigger widespread thrombosis. Moreover, deep hypothermic circulatory arrest (DHCA) complicates the problem of anticoagulation during cardiac surgery because of the combination of blood stasis and changes in enzymatic activity associated with the extreme temperature differences [56,57]. Therefore, the management of anticoagulation during CPB can be quite challenging and close cooperation with the haematology department is essential. There is no consensus in the literature as to the optimal method for assuring perioperative anticoagulation in APLS. While, monitoring anticoagulation in APLS patient during cardiac surgery remains problematic, as aPL often interfere with *in vitro *tests of hemostasis by impeding the binding of coagulation proteins to phospholipid surfaces [21]. Especially, during CPB, blood contact with extracorporeal surfaces causes stimulation of the coagulation cascade. To prevent clotting, unfractionated heparin is administered before CPB. Heparin concentrations of greater than/equal to 3 u/ml ± 1 are generally accepted as therapeutic for CPB [58], but individual patient responses to a standardized heparin dose vary. Heparin activity is assessed using the activated clotting time (ACT) which is a phospholipid dependent test and may be prolonged by LA antibodies [18]. In the normal patient, a heparin concentration of 3 uml ± 1 typically produces a kaolin ACT of more than 450 seconds. LMWH is attractive in this setting as it causes a highly predictable anticoagulant effect for a given dose, decreasing the need for monitoring [18]. Suggested alternative methods for monitoring anticoagulation during bypass in APLS patients include empirically doubling the baseline ACT or to reach an ACT twice the upper limit of normal [2], obtaining heparin concentrations by protamine titration (Hepcon) [59], performing anti-factor Xa assays, or performing heparin/ACT titration curves preoperatively to determine patient specific target ACT levels [18]. The in vitro heparin/ACT titration curve is a test of an individual patient's responsiveness to heparin. Moreover, preoperatively, anti-Xa factor activity assays can be correlated with the patient specific preoperative in vitro heparin ACT titration curve [18]. Anti-Xa monitoring is generally considered the"gold standard" laboratory measure of heparin therapy for use in situations in which the aPTT may be adversely affected [22]. By using this testing method, known concentrations of purified coagulation factor Xa and antithrombin are mixed with a sample of the patient's heparin-containing plasma [18,22]. Anti-factor Xa levels of 1.5 ± 2.0 u/ml ± 1 are considered therapeutic for CPB [18]. Postoperatively, levels greater than 1.0 u/ml ± 1 may be associated with excess blood loss [60]. When treating venous thromboembolism, the target range of anti-factor Xa activity is 0.6 ± 1.0 u/ml ± 1 [6]. However, the turnaround time for anti-factor Xa assays are currently incompatible with the time constraints of CPB [18,60]. Generally, various anticoagulation methods for dosing heparine during CPB have been performed. Especially, Sheikh et al [61] elected to empirically double the ACT to more than 999 seconds; as obtaining factor Xa or plasma heparin concentrations was considered impractical. On the other hand, East et al [22] elected to perform heparin-celite ACT titration curves preoperatively on each patient to assess the effect of APL antibodies on ACT monitoring. Based on the patient-specific titration curve, therapeutic anticoagulation (heparin concentration 3.0 U/mL) for CPB was achieved in patients at celite ACT values exceeding 550 s. Also, laboratory based anti-Xa monitoring was performed concurrently during CPB as a secondary confirmatory measure [22]. In retrospective studies, a lower incidence of recurrences (arterial and venous) episodes of thrombosis was observed in patients kept at a high intensity of oral anticoagulation [62-64] leading to the recommendation that patients with antiphospholipid antibodies who have had a documented major thrombotic event should receive life-long oral anticoagulant treatment to achieve an INR of 3.0 or higher [62,65,66]. In spite of anticoagulant treatment, recurrence of thrombosis is frequent in patients with the APLS [63,67], especially in those carrying lupus anticoagulant (LA) [68] and/or high titers of anticardiolipin antibodies [63,67]. Especially, Della Valle et al [63] showed that INR determinations obtained with a recombinant PT reagent substantially overestimate the actual degree of anticoagulation of most lupus anticoagulants patients due to interference of lupus anticoagulant IgG in PT assays carried out at low test plasma dilution, as occurs with plain and recombinant thromboplastin reagents. Also, according to the *Duration of Anticoagulation Study Group*, patients with anti-cardiolipin IgG antibodies require prolonged anticoagulation to avoid recurrences of venous thromboembolism [69]. In addition, while on oral anticoagulant treatment targeted at an INR between 2.0 and 2.85, the recurrence rate increased with the anticardiolipin antibody titter, but was not significantly different in patients with (1.32 per 100 patient-years) or without (0.6 per 100 patient-years) anti-cardiolipin antibodies [69]. Specifically, patients with primary APLS with thrombosis are treated with heparin followed by warfarin in the usual manner [18]. There is controversy over the intensity of anticoagulant therapy, the duration of treatment, and the method for measuring the INR. Recurrent thrombotic events of any type usually signal the need for life-long anticoagulation. Recurrent thrombosis while on standard intensity anticoagulant therapy dictates the use of a higher target INR at about 2,5-3,5 [70], but the INR may not correlate well with diagnosis or outcome in APLS [71]. Although the risk of thrombosis is reduced with increased anticoagulation, there is an associated increased risk of significant bleeding [18]. Protamine to antagonise heparin should be administered only in a stepwise manner or in low dose continuously intravenous, e.g. 50 mg/h [2], until the bleeding tendency slows down to an acceptable amount. Also, a scrutinised operative technique and haemostasis to avoid any unnecessary surgical bleeding site are of great importance [2]. In patients with secondary APLS and thrombosis, there is an ongoing endothelial disturbance secondary to the underlying vasculitis and the risk of recurrent thrombotic events is high [2]. Anti-platelet therapy as well as warfarin anticoagulation is indicated. Moreover, additional factors of thrombosis such as hypertension, diabetes mellitus, hyperlipidaemia should be treated optimally or avoided (e.g. smoking) [2]. Generally, antiplatelet therapy may be added but the effectiveness of low-dose aspirin or the newer antiplatelet agents is unproven. In cases where thrombosis continues despite adequate anticoagulation, additional treatment is aimed either at preventing antibody formation or reducing antibody titres and may include corticosteroids, immunosuppressive agents, i.v. immuneglobulin, or plasmapheresis. Particularly, Dorman R [18] described the perioperative course and successful treatment of a 31-yr-old woman with primary APLS requiring a mitral valve replacement; that postoperatively, developed acute global biventricular failure requiring extracorporeal membrane oxygenation support and plasmapheresis. Alternative methods for anticoagulation during CPB are essential and multiple options are available today [56]. These options include ancrod, hirudin, lepirudin, argatroban, prostacyclin, platelet IIb/IIIa inhibitors, complement inhibitors, IL-3 and bivalirudin [56,72]. Also, tissue plasminogen activator has been used successfully in one case report of APLS with ST changes and normal coronary arteries [73]. Bivalirudin is a bivalent reversible direct thrombin inhibitor and has been used safely for CPB in HIT-positive patients [56,72,74-76]. It is also successfully used during off-pump/on-pump coronary artery bypass grafting and heart transplantation [77-79]. Bivalirudin is a bivalent direct thrombin inhibitor with a short half-life of approximately 25 minutes [56]. Its 20-amino acid molecule combines a carboxy-terminal region that recognizes thrombin's fibrin (ogen)-binding site, and an amino-terminal tetrapeptide that inhibits the active site of thrombin [56]. Consequently, thrombin inhibition of bivalirudin inactivates not only fluid-phase thrombin, but also fibrin-bound thrombin [80]. Therefore, thrombus formation may be attenuated more effectively and anticoagulation produced more predictably than by heparin [56,80]. Pharmacokinetically bivalirudin is predominantly eliminated by enzymatic degradation through proteases and thrombin itself (80%) and to a lesser degree by renal clearance (20%) [81]. No reversal for bivalirudin exists, but its elimination can be enhanced by hemodialysis and hemofiltration [81,82]. The theoretical potential of aprotinin to delay bivalirudin elimination and to prolong its anticoagulant effect has not been demonstrated [56]. In one study aprotinin did not affect the elimination of bivalirudin [82]. Leissner et al [56], reported a case from a patient with APLS with massive thrombosis of right atrium. This patient underwent DHCA by using bivalirudin, for thrombectomy to avoid potential catastrophic pulmonary embolism. During CPB the ACT was restored between 500-750 sec with the initial dose of bivalirudin and during the circulatory arrest the arterial and venous lines were clamped and the CPB circuit was recirculated distal to the arterial filter back to the soft-shelled venous reservoir to avoid pump clotting [56]. The DHCA time was planned to be short and lasted 19 minutes. No visible clot formation was observed in the open-heart and bypass circuit, but in the pericardial cavity as previously attributed to increased blood stagnation [72,79]. cAPLS is caused by a generalised thrombotic storm due to excessive activation of "aPL" with consecutive multi organ failure and peripheral ischaemic lesions as digital necrosis [2]. Also, acute single organ failure (e.g. acute heart failure) representing complications of APLS should be treated similar to cAPLS. The baseline is an aggressive intravenous anticoagulation, usually heparin, later followed by oral anticoagulation with vitamin K-antagonists [2,83]. Steroids may contribute to limitation of the cytokine release and further treat the widespread vasculitis [83]. Moreover, plasmapheresis or intravenous gammaglobulins reduce the autoantibody load, improving the outcome [2,84]. The use of prostacyclin, fibrinolytics, cytotoxic drugs, splenectomy or dialysis are described but without proven advantage for survival. In Asherson and colleagues [83] most recent retrospective case series of cAPLS, only anticoagulation was associated with statistically significant increased survival. While, in previous Asherson and colleagues series [84], plasmapheresis did reduce mortality. Also, there are case reports of acute biventricular failure in APLS with necropsy findings of myocardial microvascular thrombosis [85-89]. In this situation, antithrombotic not anti-inflammatory therapy is likely to be effective [18]. A diagnosis of fulminant myocarditis could be supported by the global nature of the myocardial dysfunction and its prompt resolution. Fulminant myocarditis is associated with full recovery in over 90% of the patients who survive the event [89]. There is one case report of APLS associated postoperative fulminant myocarditis managed with immunosuppressive steroids [90]. Currently, there is some scientific rationale for adding a statin as adjunctive treatment. Statins may be beneficial in APLS patients by suppressing the inflammatory response, which is important in case of valvular lesions and vascular occlusive disease [8]. However, no clinical investigations using statins as anti-inflammatory treatment in APLS patients have been reported. Finally, specific therapeutic treatment for APLS is not available yet. The application of peptides specifically bound by anti-b2GPI antibodies thus neutralising the functional effect of the autoantibodies [91] or peptides which have a similar amino acid sequence as domain V of b2GPI thus possibly blocking putative receptors in an antagonistic manner [2,92] constitute promising therapeutic approaches.

## Outcomes of cardiac surgery in APLS patients

The literature offers widely differing estimates of morbidity and mortality associated with APLS and cardiopulmonary bypass. In a retrospective analysis of 19 patients with APLS undergoing cardiac or vascular surgical procedures, Ciocca and colleagues reported an 84.2% incidence of postoperative thrombosis or bleeding and 63.2% mortality [31]. Thirteen of the patients in these series underwent cardiac surgery. Individual case reports of cardiac surgical patients frequently describe thrombotic or haemorrhagic complications including early graft occlusion [93] haemothorax [94], pulmonary emboli, and limb ischaemia [95,96]. More optimistically, the literature includes several case reports of uneventful cardiac surgery [97,98]. A meta analysis of heart valve surgery by Gorki et al [2] demonstrated that the mortality is high with 7% early deaths and 12% late deaths after a mean follow-up period of less then 3 years. The early and late morbidity with major complications (valve related included) respectively is significant. Only 42% of the patients had an uneventful short and long-term recovery. APLS-typical processes could be explanation for the majority of postoperative problems, especially for myocardial and cerebral complications and other overt thromboembolic events. The high proportion (about 20%) of valve-related complications such as valve thrombosis is remarkable. Colli et al [99], in a recent retrospective analysis of nine patients with antiphospholipid syndrome that underwent heart valve surgery using CPB, observed high morbidity (50%) and mortality (22%) respectively. Especially, two patients died in the early postoperative period due to an acute cerebrovascular accident, four patients presented an uneventful late postoperative course and one patient experienced an ischemic stroke 5 years after mitral valve replacement and developed refractory congestive heart failure requiring heart transplantation three years postoperatively. Similarly, Berkun et al [55] presented an increased morbidity and mortality in ten patients with APLS undergoing valve replacement.

## Conclusions

APLS is one of the most commonly acquired hypercoagulable states. Minor alterations in the anticoagulation, infection, and surgical insult may trigger widespread thrombosis. The incidence of thrombosis is highest during the following perioperative periods: preoperatively during the withdrawal of warfarin, postoperatively during the period of hypercoagulability despite warfarin or heparin therapy, or postoperatively before re-establishing adequate anticoagulation. The resources to manage major complications are essential. A successful outcome requires multidisciplinary management in order to prevent thrombotic or bleeding complications and to manage perioperative anticoagulation. More work and reporting on anticoagulation management and adjuvant therapy in patients with APLS during extracorporeal circulation are necessary.

## Abbreviation list

ACS: acute coronary syndromes; ACT: activated clotting time; aCL: anticardiolipin antibodies; aPL: antiphospholipid antibodies; APLS: antiphospholipid syndrome; b2GPI: beta-2-glycoprotein-1; CPB: cardiopulmonary bypass; cAPLS: catastrophic APLS; CABG: coronary artery bypass grafting; DHCA: deep hypothermic circulatory arrest; dRVVT: diluted Russell's viper venom test; DIC: disseminated intravascular coagulation; HIT: heparin-induced thrombocytopenia; INR: international normalized ratio; ICAM-1: intracellular adhesion-molecule-1; Lp(a): lipoprotein a; LMWH: *low-molecular-weight heparin*; LA: Lupus anticoagulant; MI: myocardial infarction; aPTT: partial thromboplastin time; PCI: percutaneous coronary interventions; PS: phosphatidylserine; PL: phospholipids; PT: prothrombin time; SIRS: systemic inflammatory response syndrome; SLE: systemic lupus erythematosus; TTP: thrombotic thrombocytopenic purpura; TFPI: tissue-factor-pathway-inhibitor; t-PA: tissue type plasminogen activator; VCAM-1: vascular-cell-adhesion-molecule-1.

## Competing interests

The authors declare that they have no competing interests.

## Authors' contributions

IK, SS and NB contributed to acquisition of data, further analysis, interpretation and writing of the review. GP and JG revised critically the manuscript. EA revised critically the review and further gave the final approval for manuscript's publication. All authors read and approved the final manuscript.

## References

[B1] Miyakis S Lockshin MD Atsumi T Branch DW Brey RL Cervera R Derksen RHWM DeGroot PG Koike T Meroni PL Reber G Shoenfeld Y Tincani A Vlachoyiannopoulos PG Krilis SA International consensus statement on an update of the classification criteria for definite antiphospholipid syndrome (APS) J Thromb Haemost 2006 4 295 306 10.1111/j.1538-7836.2006.01753.x 16420554

[B2] Gorki H Malinovski V Stanbridge RDL The antiphospholipid syndrome and heart valve surgery Eur J CardioThorac Surg 2008 33 168 18 10.1016/j.ejcts.2007.11.004 18082413

[B3] Tripodi A Chantarangkul V Clerici M Negri B Galli M Mannucci PM Laboratory control of oral anticoagulant treatment by the INR system in patients with the antiphospholipid syndrome and lupus anticoagulant. Results of a collaborative study involving nine commercial thromboplastins Br J Haematol 2001 115 672 8 10.1046/j.1365-2141.2001.03178.x 11736953

[B4] Asherson RA Piette JC The catastrophic antiphospholipid syndrome: acute multiorgan failure associated with antiphospholipid antibodies: a review of 31 patients Lupus 1996 5 414 7 8902772 10.1177/096120339600500516

[B5] Hedge VAP Vivas Y Shah H Haybron D Srinivasan V Dua A Gradman A Cardiovascular surgical outcomes in patients with the antiphospholipid syndrome- a case series Heart, Lung and Circulation 2007 16 423 427 10.1016/j.hlc.2007.03.010 17611152

[B6] Gurlek A Ozdol C Pamir G Association Between Anticardiolipin Antibodies and Recurrent Cardiac Events in Patients With Acute Coronary Syndrome Int Heart J 2005 46 631 638 10.1536/ihj.46.631 16157954

[B7] Turiel M Muzzupappa S Gottardi B Evaluation of cardiac abnormalities and embolic sources in primary antiphospholipid syndrome by transesophageal echocardiography Lupus 2000 9 406 12 10.1191/096120300678828532 10981643

[B8] Weiss S Nyzio JB Cines D Detre J Milas BL Narula N Floyd TF Antiphospholipid syndrome : Intraoperative and postoperative anticoagulation in cardiac surgery J Cardiothorac Vasc Anesth 2008 22 735 9 10.1053/j.jvca.2008.01.021 18922433

[B9] Greaves M Cohen H MacHin SJ Mackie I Guidelines on the investigation and management of the antiphospholipid syndrome Br J Haematol 2000 109 704 15 10.1046/j.1365-2141.2000.02069.x 10929019

[B10] Galli M Luciani D Bertolini G Barbui T Anti-beta 2-glycoprotein I, antiprothrombin antibodies, and the risk of thrombosis in the antiphospholipid syndrome Blood 2003 102 2717 23 10.1182/blood-2002-11-3334 12816875

[B11] Oosting JD Derksen RH Bobbink IW Hackeng TM Bouma BN deGroot PG Antiphospholipid antibodies directed against a combination of phospholipids with prothrombin, protein C, or protein S: an explanation for their pathogenic mechanism? Blood 1993 81 2618 25 8166780

[B12] Galli M Comfurius P Maassen C Hemker HC de Baets MH van Breda-Vriesman PJ Barbui T Zwaal RF Bevers EM Anticardiolipin antibodies (ACA) directed not to cardiolipin but to a plasma protein cofactor Lancet 1990 335 1544 7 10.1016/0140-6736(90)91374-J 1972485

[B13] Pierangeli SS Espinola RG Liu X Harris EN Thrombogenic effects of antiphospholipid antibodies are mediated by intercellular cell adhesion molecule-1, vascular cell adhesion molecule-1, and P-selectin Circ Res 2001 88 245 50 11157679 10.1161/01.res.88.2.245

[B14] Roubey RA Tissue factor pathway and the antiphospholipid syndrome Autoimmunol 2000 15 217 20 10.1006/jaut.2000.0397 10968913

[B15] Lutters BCH Derksen RHWM Tekelenburg WL Lenting PJ Arnout J deGroot PG Dimers of beta 2-glycoprotein I increase platelet deposition to collagen via interaction with phospholipids and the apolipoprotein E receptor 2' J Biol Chem 2003 278 33831 8 10.1074/jbc.M212655200 12807892

[B16] Ma K Simantov R Zhang JC Silverstein R Hajjar KA McCrae KR High affinity binding of beta 2-glycoprotein I to human endothelial cells is mediated by annexin II J Biol Chem 2000 275 15541 8 10.1074/jbc.275.20.15541 10809787

[B17] Mackworth-Young CG Antiphospholipid syndrome: Multiple mechanisms Clin Exp Immunol 2004 136 393 401 10.1111/j.1365-2249.2004.02497.x 15147339 PMC1809064

[B18] Dornan RI Acute postoperative biventricular failure associated with antiphospholipid antibody syndrome Br J Anaesth 2004 92 748 54 10.1093/bja/aeh116 15003982

[B19] Love PE Santaro SA Antiphospholipid antibodies: anticardiolipin and the lupus anticoagulant in systemic lupus erythematosus (SLE) and in non-SLE disorders Ann Intern Med 1990 112 682 98 2110431 10.7326/0003-4819-112-9-682

[B20] Mackworth-Young C Antiphospholipin antibodies: more than just a disease marker? Immunol Today 1990 11 60 5 10.1016/0167-5699(90)90020-A 2185781

[B21] Rand JH Wu XX Andree HAM Antiphospholipid antibodies accelerate plasma coagulation by inhibiting annexin-V binding to phospholipids: a "lupus procoagulant" phenomenon Blood 1998 92 1652 60 9716593

[B22] East Chr Clements F Mathew J Antiphospholipid Syndrome and Cardiac Surgery: Management of anticoagulation in two Patients Anesth Analg 2000 90 1098 101 10.1097/00000539-200005000-00017 10781459

[B23] Meroni PL Borghi MO Raschi E Inflammatory response and the endothelium Thromb Res 2004 114 329 334 10.1016/j.thromres.2004.06.045 15507262

[B24] Cervera R Recent advances in antiphospholipid antibodyrelated valvulopathies J Autoimmun 2000 15 123 5 10.1006/jaut.2000.0405 10968897

[B25] Erdogan D Goren MT Diz-Kucukkaya R Assessment of cardiac structure and left atrial appendage functions in primary antiphospholipid syndrome: A transesophageal echocardiographic study Stroke 2005 36 592 596 10.1161/01.STR.0000154858.27353.df 15677581

[B26] Sakaguchi G Minami K Nakayama S Aortic valve replacement after previous coronary artery bypass grafting in a patient with antiphospholipid syndrome Jpn J Thorac Cardiovasc Surg 1998 46 257 9 9584474 10.1007/BF03217739

[B27] Schumacher M Eber B Dusleag J Fruhwald FM Zweiker R Pokan R Klein W Thrombosis of a prosthetic mitral valve in the anticardiolipin syndrome Dtsch Med Wochenschr 1995 120 795 8 10.1055/s-2008-1055410 7781511

[B28] Hogan WJ McBane RD Santrach PJ Plumhoff EA Oliver WC Jr Schaff HV Rodeheffer RJ Edwards WD Duffy J Nichols WL Antiphospholipid syndrome and perioperative hemostatic management of cardiac valvular surgery Mayo Clin Proc 2000 75 971 6 10.4065/75.9.971 10994834

[B29] Levine JS Branch DW Rauch J The antiphospholipid syndrome N Engl J Med 2002 346 752 63 10.1056/NEJMra002974 11882732

[B30] Nakayama M Kumon K Yahagi N Antiphospholipid antibody syndrome in a case with redo coronary artery bypass grafting under cardiopulmonary bypass Surg Today 1998 28 423 426 10.1007/s005950050155 9590711

[B31] Ciocca RG Choi J Graham AM Antiphospholipid antibodies lead to increased risk in cardiovascular surgery Am J Surg 1995 170 198 200 10.1016/S0002-9610(99)80285-2 7631930

[B32] Jorgensen P Hansen PR Antiphospholipid antibodies and ischaemic heart disease J Intern Med 1993 233 291 293 10.1111/j.1365-2796.1993.tb00990.x 8450299

[B33] Tektonidou MG Ionnidis JPA Boki KA Vlachoyiannopoulos PG Moutsopoulos HM Prognostic factors and clustering of serious clinical outcomes in antiphospholipid syndrome Q J Med 2000 93 523 30 10.1093/qjmed/93.8.523 10924534

[B34] Harris EN Hughes GR Gharav AE Antiphospholipid antibodies: an elderly statesman dons new garments J Rheumatol 1987 14 Suppl 13 208 13 3112376

[B35] Shapiro SS Thiagarajan P Lupus anticoagulants Prog Hemost Thromb 1982 6 263 85 6820164

[B36] Erkan D Lockshin Non-criteria manifestations of antiphospholipid syndrome Lupus 2010 19 4 424 7 10.1177/0961203309360545 20353981

[B37] Asherson RA Cervera R Piette JC Shoenfeld Y Espinosa G Petri MA Lim E Lau TC Gurjal A Jedryka-Góral A Chwalinska-Sadowska H Dibner RJ Rojas-Rodríguez J García-Carrasco M Grandone JT Parke AL Barbosa P Vasconcelos C Ramos-Casals M Font J Ingelmo M Catastrophic antiphospholipid syndrome: clues to the pathogenesis from a series of 80 patients Med Baltim 2001 80 355 77 10.1097/00005792-200111000-00002 11704713

[B38] Greaves M Cohen H MacHin SJ Mackie I Guidelines on the investigation and management of the antiphospholipid syndrome Br J Haematol 2000 109 704 15 10.1046/j.1365-2141.2000.02069.x 10929019

[B39] Triplett DA Coagulation assays for the lupus anticoagulant: review and critique of current methodology Stroke 1992 23 Suppl I 11 4 1561667

[B40] Eby C Standardization of APTTreagents for heparin therapy monitoring: urgent or fading priority? Clin Chem 1997 43 1105 7 9216444

[B41] Arvieux J Darnige L Hachulla E Roussel B Bensa JC Coulomb MG Species specificity of anti-beta 2 glycoprotein I autoantibodies and its relevance to anticardiolipin antibody quantitation Thromb Haemost 1996 75 725 30 8725713

[B42] Bertolaccini ML Atsumi T Koike T Hughes GRV Khamashta MA Antiprothrombin antibodies detected in two different assay systems. Prevalence and clinical significance in systemic lupus erythematosus Thromb Haemost 2005 93 289 97 15711745 10.1160/TH04-06-0382

[B43] Hughes GR The antiphospholipid syndrome: ten years on Lancet 1993 342 341 4 10.1016/0140-6736(93)91477-4 8101587

[B44] Eber B Kronberger-Schaffer E Brusee H Anticardiolipin antibodies are no marker for survived myocardial infarction Klin Wochenschr 1990 68 594 6 10.1007/BF01660956 2376955

[B45] Ludia C Domenico P Monia C Antiphospholipid antibodies: a new risk factor for restenosis after percutaneous transluminal coronary angioplasty? Autoimmunity 1998 27 141 8 10.3109/08916939809003861 9609131

[B46] Chiarugi L Prisco D Antonucci E Lipoprotein (a) and anticardiolipin antibodies are risk factors for clinically relevant restosis after elective balloon precutaneus transluminal coronary angioplasty Atherosclerosis 2001 154 129 35 10.1016/S0021-9150(00)00439-1 11137091

[B47] Sharma S Malhotra A Sharma YP Pandhi P Malhotra S Nageswari KS Shafiq N Venkateshan SP Kaur R Association of anticardiolipin antibodies levels with instent restenosis in patients with coronary artery disease Indian J Physiol Pharmacol 2008 52 3 288 92 19552061

[B48] Bili A Moss AJ Francis CW Zareba W Watelet LF Sanz I Anticardiolipin antibodies and recurrent coronary events: a prospective study of 1150 patients. Thrombogenic Factors, and Recurrent Coronary Events Invertigators Circulation 2000 102 1258 63 10982540 10.1161/01.cir.102.11.1258

[B49] Zuckerman E Toubi E Shiran A Anticardiolipin antibodies and acute myocardial infarction in non-systemic lupus erythematosus patients: a controlled prospective study Am J Med 1996 104 381 6 10.1016/S0002-9343(96)00226-4 8873508

[B50] Hamsten Ai Norberg R Bjorkholm M de Faire U Holm G Antibodies to cardiolipin in young survivors of myocardial infarction: an association with recurrent cardiovascular events Lancet 1986 1 113 6 10.1016/S0140-6736(86)92258-0 2867345

[B51] Sletnes KE Smith P Abdelnoor N Arnesen H Wisloff F Antiphospholipid antibodies after myocardial infarction and their relation to mortality, reinfarction, and non-hemorrhagic stroke Lancet 1992 339 451 3 10.1016/0140-6736(92)91057-F 1346819

[B52] Cortellaro M Boschetti C Cardillo M Barbui T Antiphospholipid antibodies in patients with previous myocardial infaction Lancet 1992 339 929 30 10.1016/0140-6736(92)90966-7 1348318

[B53] Phadke KV Phillips RA Clarke DT Jones M Naish P Carson P Anticardiolipin antibodies in ischemic heart disease: marker or myth? Br Heart J 1993 69 391 4 10.1136/hrt.69.5.391 8518060 PMC1025099

[B54] Warkentin TE Aird WC Rand JH Platelet-Endothelial Interactions: Sepsis, HIT, and Antiphospholipid Syndrome Hematology 2003 1 497 519 10.1182/asheducation-2003.1.497 14633796

[B55] Berkun Y Elami A Meir K Increased morbidity and mortality in patients with antiphospholipid syndrome undergoing valve replacement surgery J Thorac Cardiovasc Surg 2004 127 414 420 10.1016/j.jtcvs.2003.07.016 14762349

[B56] Leissner K Ketchedjian A Crowley R Deep hypothermic circulatory arrest and Bivalirudin use in a patient with heparin-induced thrombocytopenia and antiphospholipid syndrome J Card Surg 2007 22 78 82 10.1111/j.1540-8191.2007.00351.x 17239224

[B57] Green JA Spiess BD Current status of antifibrinolytics in cardiopulmonary bypass and elective deep hypothermic circulatory arrest Anesthesiol Clin North America 2003 21 527 551 10.1016/S0889-8537(03)00042-7 14562564

[B58] Despotis GJ Joist JH Hogue CW jr The impact of heparin concentration and cativated clotting time monitoring on blood conservation J Thorac Cardiovasc Surg 1995 110 46 54 10.1016/S0022-5223(05)80008-X 7609568

[B59] Hogan WJ McBane RD Santrach PJ Antiphospholipid syndrome and perioperative haemostatic management of cardiac valvular surgery Mayo Clin Proc 2000 75 971 6 10.4065/75.9.971 10994834

[B60] East CJ Clements F Mathew J Slaughter TF Antiphospholipid syndrome and cardiac surgery: management of anticoagulation in two patients Anesth Analg 2000 90 1098 101 10.1097/00000539-200005000-00017 10781459

[B61] Shiekh F Lechowicz A Setlur R Recognition and management of patients with antiphospholipid antibody syndrome undergoing cardiac surgery J Cardiothor Vasc Anesth 1997 11 764 6 10.1016/S1053-0770(97)90173-7 9327321

[B62] Khamashta MA Cuadrado MJ Mujic F Taub NA Hunt BJ Hughes GRV The management of thrombosis in the antiphospholipid-antibody syndrome N Engl J Med 1995 332 993 7 10.1056/NEJM199504133321504 7885428

[B63] Della Valle P Crippa L Garlando A-M Interference of lupus anticoagulants in prothrombin time assays: implications for selection of adequate methods to optimize the management of thrombosis in antiphospholipid-antibody syndrome Haematologica 1999 84 1065 1074 10586206

[B64] Rivier G Herranz MT Khamashta MA Hughes GRV hrombosis and antiphospholipid syndrome: a preliminary assessment of three antithrombotic treatments Lupus 1994 3 85 90 10.1177/096120339400300205 7920619

[B65] Asherson RA Cervera R Merrill JT Erkan D Antiphospholipid antibodies and the antiphospholipid syndrome: clinical significance and treatment Semin Thromb Hemost 2008 34 3 256 66 10.1055/s-0028-1082269 18720305

[B66] Lockshin MD Answers to the antiphospholipid-antibody syndrome? N Engl J Med 1995 332 1025 7 10.1056/NEJM199504133321510 7885409

[B67] Finazzi G Brancaccio V Moia M Natural history and risk factors for thrombosis in 360 patients with antiphospholipid antibodies. A five-year prospective study from the Italian registry Am J Med 1996 100 530 6 10.1016/S0002-9343(96)00060-5 8644765

[B68] Ginsberg JS Wells PS Brill-Edwards P Antiphospholipid antibodies and venous thromboembolism Blood 1995 86 3685 91 7579334

[B69] Schulman S Svenungsson E Granqvist S The Duration of Anticoagulation Study Group Anticardiolipin antibodies predict early recurrence of thromboembolism and death among patients with venous thromboembolism following anticoagulant therapy Am J Med 1998 104 332 8 10.1016/S0002-9343(98)00060-6 9576405

[B70] Haemostasis and Thrombosis Task Force. Guidelines on the investigation and management of the antiphospholipid syndrome Br J Haematol 2000 109 704 15 10.1046/j.1365-2141.2000.02069.x 10929019

[B71] Tripodi A Chantarangkul V Clerici M Negri B Galli M Mannucci PM Laboratory control of oral anticoagulant treatment by the INR system in patients with the antiphospholipid syndrome and lupus anticoagulant. Results of a collaborative study involving nine commercial thromboplastins Br J Haematol 2001 115 672 8 10.1046/j.1365-2141.2001.03178.x 11736953

[B72] Gordon G Rastegar H Schumann R Successful use of bivalirudin for cardiopulmonary bypass in a patient with heparin-induced thrombocytopenia J Cardiothorac Vasc Anesth 2003 17 632 635 10.1016/S1053-0770(03)00210-6 14579220

[B73] Harpaz D Glikson M Side Y Hod H Successful thrombolytic therapy for acute myocardial infarction in a patient with the antiphospholipid antibody syndrome Am Heart J 1991 122 1492 4 10.1016/0002-8703(91)90604-G 1951025

[B74] Merry AF Bivalirudin, blood loss, and graft patency in coronary artery bypass surgery Semin Thromb Hemost 2004 30 337 346 10.1055/s-2004-831046 15282656

[B75] Koster A Yeter R Buz S Assessment of hemostatic activation during cardiopulmonary bypass for coronary artery bypass grafting with bivalirudin: Results of a pilot study J Thorac Cardiovasc Surg 2005 129 1391 1394 10.1016/j.jtcvs.2004.09.016 15942583

[B76] Clayton SB Acsell JR Crumbley AJ cardiopulmonary bypass with bivalirudin in type II heparin- nduced thrombocytopenia Ann Thorac Surg 2004 78 2167 2169 10.1016/S0003-4975(03)01471-1 15561064

[B77] Merry AF Raudkivi PJ Middleton NG Bivalirudin versus heparin and protamine in off-pump coronary artery bypass surgery Ann Thorac Surg 2004 77 925 931 10.1016/j.athoracsur.2003.09.061 14992900

[B78] Mann MJ Tseng E Ratcliffe M Use of bivalirudin, a direct thrombin inhibitor, and its reversal with modified ultrafiltration during heart transplantation in a patient with heparin-induced thrombocytopenia J Heart Lung Transplant 2005 24 222 225 10.1016/j.healun.2003.11.401 15701441

[B79] Greinacher A The use of direct thrombin inhibitors in cardiovascular surgery in patients with heparin-induced thrombocytopenia Semin Thromb Hemost 2004 30 315 327 10.1055/s-2004-831044 15282654

[B80] Weitz JI Bates SM New anticoagulants J Thromb Haemost 2005 3 1843 1853 10.1111/j.1538-7836.2005.01374.x 16102051

[B81] Koster A Spiess B Chew DP Effectiveness of bivalirudin as a replacement for heparin during cardiopulmonary bypass in patients undergoing coronary artery bypass grafting Am J Cardiol 2004 93 356 359 10.1016/j.amjcard.2003.10.021 14759391

[B82] Dyke CM Koster A Veale JJ Preemptive use of Bivalirudin for urgent on-pump coronary artery bypass grafting in patients with potential heparin-induced thrombocytopenia Ann Thorac Surg 2005 80 299 303 10.1016/j.athoracsur.2004.08.037 15975385

[B83] Asherson RA Cervera R Piette JC Catastrophic antiphospholipid syndrome: clues to the pathogenesis from a series of 80 patients Medicine 2001 80 355 77 10.1097/00005792-200111000-00002 11704713

[B84] Asherson RA Cervera R Piette JC Catastrophic antiphospholipid syndrome: Clinical and laboratory features of 50 patients Medicine 1998 77 195 207 10.1097/00005792-199805000-00005 9653431

[B85] Asherson R Cervera R Antiphospholipid antibodies and the heart. Lessons and pitfalls for the cardiologist Circulation 1991 84 920 3 1860234 10.1161/01.cir.84.2.920

[B86] Menon G Allt-Graham J Anaesthetic implications of the anticardiolipin antibody syndrome Br J Anaesth 1993 70 587 90 10.1093/bja/70.5.587 8318336

[B87] Brown JH Doherty CC Allen DC Morton P Fatal cardiac failure due to myocardial microthrombi in systemic lupus erythematosus BMJ 1988 296 1505 10.1136/bmj.296.6635.1505 3134088 PMC2546021

[B88] McCarthy RE Boehmer JP Hruban RH Long-term outcome of fulminant myocarditis as compared with acute (nonfulminant) myocarditis N Engl J Med 2000 342 690 5 10.1056/NEJM200003093421003 10706898

[B89] Murphy JJ Leach IA Findings at necropsy in the heart of a patient with anticardiolipin syndrome Br Heart J 1989 62 61 4 10.1136/hrt.62.1.61 2757875 PMC1216732

[B90] Nagappan R Lodge RS Acute autoimmune cardiomyopathy in primary antiphospholipid antibody syndrome Anaesth Intens Care 2002 30 226 9 10.1177/0310057X0203000219 12002935

[B91] Sherer Y Snoenfeld Y Antiphospholipid syndrome: insights from animal models Curr Opin Hematol 2000 7 321 4 10.1097/00062752-200009000-00011 10961584

[B92] Vega Ostertag M Liu X Henderson V Pierangeli SS A peptide that mimics the Vth region of beta-2-glycoprotein I reverses antiphospholipid-mediated thrombosis in mice Lupus 2006 15 358 65 10.1191/0961203306lu2315oa 16830882

[B93] Morton KE Gavaghan TP Krilis SA Coronary artery bypass graft failure -An autoimmune phenomenon? Lancet 1986 ii 1353 6 10.1016/S0140-6736(86)92004-0 2878224

[B94] Myers G Hirsch G Double valve replacement in a patient with anticardiolipin antibody syndrome Perfusion 1999 14 397 401 10499657 10.1177/026765919901400512

[B95] Asherson R Weinberger A Kinsley R Radial artery occlusion in primary antiphospholipid syndrome after aortic valve replacement S Afr Med J 1995 85 1042 8596982

[B96] Sheikh F Lechowicz A Selut R Rauch A Dunn H Recognition and management of patients with antiphospholipid antibody syndrome undergoing cardiac surgery J Cardiothorac Vasc Anesth 1997 11 764 6 10.1016/S1053-0770(97)90173-7 9327321

[B97] Ando M Takamoto S Okita Y Operation for chronic pulmonary thromboembolism accompanied by thrombophilia in 8 patients Ann Thorac Surg 1998 66 1919 24 10.1016/S0003-4975(98)00910-2 9930469

[B98] Ducart AR Collard EL Osselaer JC Broka SM Eucher PM Joucken KL Management of anticoagulation during cardiopulmonary bypass in a patient with a circulating lupus anticoagulant J Cardiothorac Vasc Anesth 1997 11 878 9 10.1016/S1053-0770(97)90125-7 9412889

[B99] Colli A Mestres CA Espinosa G Plasín MA Pomar JL Font J Cervera R Heart valve surgery in patients with the antiphospholipid syndrome: analysis of a series of nine cases Eur J Cardiothorac Surg 2010 37 1 154 8 10.1016/j.ejcts.2009.06.046 19699100

